# A pilot exploration of the relationships between optimism, affect, and cardiovascular reactivity

**DOI:** 10.3389/fpsyg.2023.1233900

**Published:** 2023-10-16

**Authors:** Cesar Parra-Gaete, Carlos Hermosa-Bosano

**Affiliations:** Grupo de Investigación Bienestar, Salud y Sociedad, Escuela de Psicología y Educación, Universidad de Las Américas, Quito, Ecuador

**Keywords:** optimism and health, cardiovascular reactivity, affect, blunted reactivity, LOT-R, PANAS inventory

## Abstract

**Introduction:**

Cardiovascular reactivity refers to changes in blood pressure and heart rate in response to internal or external stimuli. Previous research has shown that excessively high and low cardiovascular reactivity are associated with an increased risk of cardiac problems. Dispositional optimism has been associated with numerous health benefits, including better cardiovascular responses to stressors, and reduced mortality risk. Conversely, pessimism has been associated with negative health outcomes and worse cardiovascular reactivity to stress. Mood, comprising positive and negative affect, can significantly impact psychological adjustment and physical health. Therefore, it is important to consider mood as a potential confounding variable in the link between optimism and cardiovascular reactivity. The study hypothesized that optimism and pessimism would still influence cardiovascular reactivity even when mood variables were controlled for.

**Methods:**

A within-subjects correlational design with 107 young adult participants was used. Sociodemographic and clinical questionnaires were administered to collect information on participants’ characteristics. The Dispositional Optimism Scale (LOT-R) and the Positive and Negative Affect Scale (PANAS) were used to assess participants’ levels of optimism, pessimism, and mood. Measures of cardiovascular reactivity, including systolic blood pressure (SBP), diastolic blood pressure (DBP), and heart rate (HR), were taken during a stressor task (PASAT).

**Results:**

There is a moderate positive correlation between dispositional optimism and positive affect, while pessimism demonstrated a moderate positive association with negative affect. Linear regression analyses were conducted, controlling baseline reactivity variables, gender, and body mass index. The results showed that pessimism had a significant negative effect on SBP reactivity, suggesting that higher levels of pessimism decreased SBP response. Optimism had a significant positive effect on DBP reactivity, while pessimism had a significant negative effect.

**Discussion:**

Overall, these results suggest that dispositional optimism and pessimism are related to cardiovascular reactivity, even after controlling for positive and negative affect. Pessimism was associated with lower SBP reactivity, while both optimism and pessimism influenced DBP reactivity. These findings are consistent with previous research indicating that optimism enables more effective stress management during challenging events, whereas pessimism can serve as a risk factor, heightening the likelihood of experiencing future cardiac issued caused by blunted cardiovascular reactivity.

## Introduction

Cardiovascular disorders, including conditions like coronary heart disease (CHD) and strokes, continue to represent a significant health challenge on a global scale, contributing to approximately 25% of all reported fatalities worldwide (Roth et al., 2018). In addition to well-established risk factors such as smoking, obesity, diabetes, a family history of heart disease, and low levels of physical activity (Helfand et al., 2009), there is a growing body of literature suggesting that psychological factors may significantly contribute to CHD, including cardiovascular reactivity (CVR) which is characterized by changes in blood pressure, heart rate, and cortisol due to internal or external stimulus. CVR is measured through systolic blood pressure (SBP), diastolic blood pressure (DBP) and heart rate (HR) reactions to the presence of stressors (Allen, 2013). Research from over 30 years (Manuck and Schaefer, 1978; Manuck and Garland, 1980; Sherwood et al., 1990) has suggested that reactivity tends to be highly to moderately stable among young, healthy individuals with no family history of heart problems (Veit et al., 1997; Pokroy et al., 2001; Rutledge and Linden, 2003). In addition, it has been used as a predictor of future pathologies. Specifically, the cardiac reactivity hypothesis proposes that maladaptive CVR responses to psychological stress, which could be exaggerated, prolonged, or diminished, might promote the development of cardiovascular disease (Phillips et al., 2013).

This hypothesis has gained substantial support from prospective studies linking heightened reactivity to stress with negative cardiovascular outcomes, including hypertension, atherosclerosis, and even mortality (Carroll et al., 2012; Brinkmann and Franzen, 2017; Ginty et al., 2017; Franzen et al., 2019; Turner et al., 2020). On the opposite end of CVR, weakened stress responses can indicate cardiovascular dysregulation (Carroll et al., 2017), and are also associated with adverse health-related consequences (Phillips and Hughes, 2011), including obesity (Carroll et al., 2008), impaired cognitive function (Ginty et al., 2012), heightened stress levels, substance abuse (Brown et al., 2019), and elevated carotid intima-media thickness (Ginty et al., 2016), which in the long term has been linked to an increased HR as the heart has to work harder to circulate blood (Brown et al., 2019). Studies have linked variations in CVR to stress with brain areas such as the limbic system, the prefrontal area, and the hypothalamus (Lovallo and Gerin, 2003). Therefore, it appears that maladaptive stress responses may indicate dysfunction in the fronto-limbic system, which can lead to behavioral and motivational issues, providing a possible explanation for the aforementioned conditions (Gianaros et al., 2005; Ginty et al., 2013; Carroll et al., 2017; O’Riordan et al., 2023).

Research into maladaptive reactivity patterns has highlighted stressful life events as factors connected to reduced responses. In a meta-analysis of 161 articles from 1988 to 2008, individuals experiencing negative life events were found to display an abnormally diminished cardiovascular response when subjected to psychological stress (Chida and Hamer, 2008). This suggests that the challenges of everyday life could contribute to a weakened cardiovascular response to immediate stressors. These findings align with the concept of allostatic load, where continuous exposure to chronic stress disrupts the coordinated functioning of mind–body regulatory systems, ultimately increasing susceptibility to illness (McEwen, 2005).

Furthermore, emotional states and personality traits such as depression, low motivation, type D personality, and neuroticism have been shown to negatively impact CVR in response to acute stress (Denollet, 2005; Chida and Steptoe, 2008). Conversely, positive factors like social support, positive affect and gratitude have been identified as stress buffers, offering cardiovascular protection in young people (Riordan et al., 2019; Gallagher et al., 2020; McMahon et al., 2020). Considering this, it becomes pertinent to inquire whether dispositional optimism, a cornerstone of positive psychology, might yield analogous outcomes in the same population.

Dispositional optimism can be defined as the inclination to hold positive expectations toward forthcoming events (Rand et al., 2020). Conversely, its counterpart, pessimism, involves holding negative perspectives about upcoming situations circumstances (Ortiz et al., 2016). These disparities in outlooks influence emotions, thoughts, and behaviors in response to upcoming events. Pessimistic individuals tend to attribute negative outcomes to internal, general, and enduring causes, whereas optimistic individuals attribute such outcomes to external, momentary, and non-specific factors (Gavín-Chocano et al., 2023). Consequently, one’s optimistic or pessimistic stance shapes their interaction with the environment, affecting their motivation and efforts in navigating life’s challenges (Scheier and Carver, 1987).

Given its impact on motivation and behavior, dispositional optimism has been closely linked to heightened individual well-being, perceived health, reduced all-cause mortality, longer life spam, healthy habits in young adults and older people (Boehm et al., 2018; Lee et al., 2019; Levine et al., 2021; Krittanawong et al., 2022). In the realm of education, it emerges as a strong predictor of academic achievement, emotional resilience, cognitive adjustment, lower levels of anxiety and depression among undergraduate students (Rand et al., 2020). The explanation for these results lies in the fact that positive expectations empower individuals to channel their energy and focus toward addressing the source of stress, thereby enhancing their stress coping abilities. Additionally, the motivation to take action that optimism creates may also lead to the reinforcement of adaptive behaviors (Rezaei and Bahadori Khosroshahi, 2018). Conversely, low levels of optimism give rise to negative or pessimistic expectations that turn attention inward, fostering feelings of hopelessness and avoidance behaviors (Baumgartner et al., 2018).

Furthermore, dispositional optimism has been correlated with more adaptive cardiovascular responses to stress-inducing stimuli in young adults (Bajaj et al., 2019). Studies have indicated a connection between optimism, decreased cardiovascular events, and lower mortality risk (Krittanawong et al., 2022). This could be attributed to the healthier behaviors exhibited by optimistic individuals, such as better sleep, increased exercise, and improved dietary habits (Steptoe, 2019). Alternatively, it might be due to the heightened stress response and improved emotional regulation demonstrated by optimistic individuals (Dieterich et al., 2020; Muazzam et al., 2021). In contrast, pessimism has been associated with poorer cardiovascular health and an elevated mortality risk (Pänkäläinen et al., 2016; Felt et al., 2020; Whitfield et al., 2020), as well as worse cardiovascular reactivity to stressors (Krittanawong et al., 2023). This might stem from the fact that pessimistic individuals struggle with effective stress management and tend to approach challenges with a defeatist attitude instead of actively problem-solving (Baumgartner et al., 2018).

However, studies have yet to thoroughly explore the potential impact of mood on the link between optimism and cardiovascular reactivity. Accounting for this variable is crucial, given that mood has been associated with both psychological adjustment and physical health (Pressman et al., 2019). Mood is commonly categorized into positive and negative affect. Positive affect signifies a state of heightened energy, enthusiasm, and focus, whereas negative affect comprises feelings of anger, subjective discomfort, and low mood states (Bayrami et al., 2012). Research indicates that positive affect is beneficial for cardiovascular health, acting as a protective factor against cardiovascular diseases and excessive reactivity, while negative affect is associated with abnormally low cardiovascular reactivity (Davidson et al., 2010; Sin, 2016; Kubzansky et al., 2018; Levine et al., 2021). There are different hypotheses about why positive and negative affect have an impact on health, such as the idea that individuals with higher positive affect tend to sleep better and engage in more physical activity, both variables related to better cardiac reactivity (Pressman et al., 2019). In the psychological realm, positive affect has been linked to a stronger social support network and better psychological well-being (Chida and Steptoe, 2008). Conversely, negative affect has been associated with loneliness and psychological distress; loneliness, particularly, has been linked to exhibiting atypically low cardiovascular reactivity especially in young adults (Brown et al., 2018, 2019).

Given these existing findings, the present study aimed to explore the connection between dispositional optimism and cardiovascular reactivity while accounting for the influence of positive and negative affect in young adults. The objective is to determine whether a relationship between optimism and cardiovascular reactivity persists even after controlling for these variables, or if the observed effect is mediated by participants’ mood states. The hypothesis posits that even with positive and negative affect controlled for, dispositional optimism and pessimism will still influence cardiovascular reactivity.

## Materials and methods

### Design

A within-subjects correlational design was used with dispositional optimism and pessimism as the main predictor variables. Control variables included positive and negative affect, body mass index, gender, and baseline cardiovascular reactivity. The dependent variables were cardiac measures including systolic blood pressure (SBP), diastolic blood pressure (DBP), and heart rate (HR).

### Participants

One hundred and seven young adults participated in this study, 69 females and 38 males. The participants were recruited from various local universities. Their ages ranged from 18 to 32 years old, with a mean age of 22.24 (*SD* = 3.27). The average body mass index (BMI) was 23.77 kg/m^2^ (*SD* = 4.23). Regarding lifestyle factors, 24% of participants reported being smokers, and 16% reported vaping. Most participants (79%) reported having had COVID-19 before participating in the study. All participants were college students. Participants were recruited through internal advertisements on the campus, by word of mouth and by a course of credit system within the university.

Exclusion criteria were applied, including individuals with a history of cardiac pathology or hypertension in their immediate family, individuals with intellectual disabilities, pregnant women, and those with any current medical or psychological illnesses. Additionally, participants were asked to refrain from consuming alcoholic beverages or exercising in the 24 h prior to the evaluation. Similarly, they were instructed not to smoke or consume caffeine in the 2 h leading up to the evaluation, as these behaviors can affect blood pressure (Potter et al., 1986; Somers et al., 1991). All participants signed an informed consent form, which included information about the study’s objectives and clarification that the research did not represent any medical risks.

### Instruments

#### Sociodemographic and clinical questionnaire

We administered a virtual questionnaire using Google Forms. This survey covered details such as participants’ sex, age, smoking or vaping habits, and any prior COVID-19 diagnoses. Additionally, we assessed other inclusion criteria variables using self-report items. These variables encompassed a history of cardiac ailments or hypertension within their immediate family, the presence of intellectual disability, pregnancy status, and the existence of any concurrent medical conditions.

#### The paired auditory serial addition test

Devised by Gronwall (1977), is a mental arithmetic assessment that entails the presentation of a series of arbitrary numbers ranging from 1 to 9. Participants are tasked with adding each new digit to the previous one. In the context of the experimental sessions, this test was employed as the stressor stimulus. Its reliability in inducing controlled changes in cardiovascular activity within laboratory environments has been established, and it has been widely utilized in numerous studies related to CVR (Carroll et al., 2008; Brindle et al., 2017; Brown et al., 2018; Gallagher et al., 2018; Chauntry et al., 2019; Riordan et al., 2019; John-Henderson et al., 2020; McMahon et al., 2020).

#### Dispositional Optimism Scale (LOT-R)

Developed by Scheier et al. (1994), this scale assesses an individual’s degree of optimism and pessimism using six 5-point Likert scale items. Higher scores indicate greater dispositional optimism. In this study, we utilized the Spanish version of Remor et al. (2006),which demonstrates an internal consistency of 0.72. In our research, the scale showed a reliability value of 0.71.

#### Positive and Negative Affect Scale

Developed by Watson et al. (1988), this scale measures an individual’s propensity to approach life in a positive manner and was used to assess participants’ emotional states. It consists of 10 items each for positive affect and negative affect, rated on a Likert-type scale ranging from 1 to 5. We utilized the Spanish version created by Sandín et al. (1999) for our study. Previous research has reported internal consistencies of 0.9 for the positive affect subscale and 0.91 for the negative affect subscale (Sandín and Chorot, 2017). In our current study, we observed reliability values of 0.86 for positive affect and 0.9 for negative affect.

#### Measurement of cardiovascular response

The participants’ systolic blood pressure (SBP), diastolic blood pressure (DBP), and heart rate (HR) were measured using the A&D BP model (UA-651) medical monitor. After instructing participants to relax, we fitted them with this arm-based device. Studies utilizing this device have consistently yielded reliable measurements (Benetti et al., 2014).

### Procedures

Before participating in the experimental session, volunteers were required to complete a sociodemographic and clinical questionnaire. After confirming their eligibility based on the inclusion criteria, they were directed to access the Lot-R and the Positive and Negative Affect Scale (PANAS) online. Once these assessments were completed, they were scheduled for the experimental session.

For the experimental phase, participants who had agreed to take part attended a 45-min sampling session at the psychology and education laboratory, scheduled from 10:00 AM to 4:00 PM. It’s worth noting that the laboratory’s windows and curtains were closed to minimize distractions, with appropriate lighting and a controlled temperature of 19 degrees Celsius maintained. During the session, the study’s objectives were reiterated, and participants signed informed consent forms. Subsequently, their weight and height were measured to calculate their body mass index (BMI). Participants were then seated in front of a desk with a computer, with their feet placed within small, low cardboard box (36 cm wide, 40 cm long, and 10 cm high) to prevent involuntary movements, a practice validated by Gallagher et al. (2018) and Jennings et al. (2007). The researcher and an assistant affixed the heart monitor to the participant’s left arm, performed an initial heart rate measurement to ensure proper functionality and participant comfort, and positioned themselves on the opposite side of the table. Throughout this setup process, the researchers restated the study’s objectives, addressed any participant queries, and provided magazines for reading during the waiting period. Following a twenty-minute rest interval, three baseline measurements of cardiac activity were recorded over two-minute intervals to establish a cardiovascular baseline.

After the baseline measurements, participants were briefed on the paired auditory serial addition test (PASAT) test, including a 10-number practice trial to ensure comprehension of the instructions and evaluate participants’ ability to accurately hear the computed-generated number sequences. It is important to emphasize that the volume of the stressor recording remained consistent for all participants, and none reported difficulties in hearing the recording. Participants were reminded that the task was evaluative, requiring verbal provision of correct answers, and that they could withdraw from the study at any time. Subsequently, the stress-inducing stimulus (PASAT) was introduced via computer recording, with cardiovascular reactivity measurements taken at two-minute intervals. Participants remained seated while the researcher and a research assistant silently recorded cardiac measurements and noted participant responses using a red pen. These conditions were designed to elevate participants’ stress levels.

Upon task completion, participants rated the task’s difficulty and their stress levels during the task, and their motivation. Following this, three additional cardiac measurements were taken at two-minute intervals to confirm if SBP, DBP, and HR had returned to baseline levels. The heart monitor was then removed, participants were asked to remove their feet from the cardboard box, and they were thanked and guided out of the laboratory by the research assistant.

### Data analysis

Before conducting the analyses, the data underwent a normality assessment using the Wilcoxon test to confirm that the assumptions of the statistical tests were met. The initial analysis involved testing for differences between the qualitative sociodemographic variables and the reactivity variables. This was done to explore potential associations between sociodemographic factors and cardiovascular reactivity. Following this, correlation analyses were performed to assess the relationships between the test results and the reactivity variables. The aim of this analysis was to identify any significant associations between the two sets of variables. Next, t-tests were conducted to compare baseline and on-task cardiac reactivity, determining whether there were significant changes in cardiovascular response induced by the stressor stimulus. Finally, stepwise linear regressions were carried out. In step 1, sociodemographic variables were included as predictors, and in step 2, the predictor variables (optimism, pessimism, positive affect, negative affect) were introduced. All statistical analyses were performed using the R statistical program (R Core Team, 2023).

## Results

The descriptive statistics for the Lot-R, pessimism, stress, and reactivity variables can be found in Table 1. A statistically significant moderate positive relationship was found between optimism and positive affect (*r* = 0.5, *p* < 0.001). Additionally, a small negative relationship between optimism and negative affect was found (*r* = −0.23, *p* = 0.019). Regarding pessimism, only a statistically significant moderate positive relationship was found with negative affect (*r* = 0.51, *p* < 0.001). A series of dependent sample t-tests confirmed an increase in cardiovascular reactivity for the following measures: SBP (*t* = 9.53, *p* < 0.0001, Cohen’s *d* = 0.92), DBP (*t* = 11.41, *p* < 0.0001, Cohen’s *d* = 0.61) and HR (*t* = 9.4, *p* < 0.0001, Cohen’s *d* = 0.91). Furthermore, there was a statistically significant increase in self-reported stress (*t* = 5.19, *p* < 0.0001, Cohen’s *d* = 0.5) and anxiety (*t* = 7.57, *p* < 0.0001, Cohen’s *d* = 0.73).

**Table 1:** Descriptive statistics cardiovascular and psychological variables.

	$\bar{X}$	SD	Mdn	Min.	Max.
Baseline SBP	110.05	10.34	109	87.5	140.5
Baseline DBP	70.84	7.62	70.75	54	95
Baseline DBP	78.62	11.49	77.5	51	109.5
Stress task SBP	116.41	12.42	115.67	91	161
Stress task DBP	76.77	8.08	76.5	58	105
Stress task HR	84.12	11.06	84	62.67	109.67
Pre-task stress	4.1	1.45	4	0	6
Post-task stress	4.65	1.25	5	0	6
Pre-task anxiety	3.8	1.55	4	0	6
Post-task anxiety	4.74	1.15	5	0	6
Optimism	9.94	2.47	10	3	15
Pessimism	7.91	2.09	8	3	13
Positive affect	30.94	6.74	31	15	50
Negative affect	23.55	8.36	21	10	48

No statistically significant differences were found in optimism, pessimism, positive and negative affect based on gender. Regarding reactivity variables, it was found that men presented higher baseline SBP than women (*t* = 5.63, *p* < 0.0001, Cohen’s *d* = 1.23, $\bar{m}$ = 117.37, $\bar{w}$ = 106. 45), however women scored higher in baseline HR (*t* = 2.32, *p* = 0.02, Cohen’s *d* = 0.47, $\bar{m}$ = 74.78, $\bar{w}$ = 80.21), stress (*t* = 4.12, *p* = 0.0001, Cohen’s *d* = 0.98, $\bar{m}$ = 3.24, $\bar{w}$ = 4.54) and perceived anxiety (*t* = 5.17, *p* < 0.0001, Cohen’s *d* = 1.16, $\bar{m}$ = 2.79, $\bar{w}$ = 4.36). No effect of the variables smoking, vaping and past COVID-19 history on reactivity scores were found. However, a moderate statistically significant relationship was found between body mass index, baseline SBP (*r* = 0.41, *p* < 0.001) and DBP (*r* = 0.44, *p* < 0.001) blood pressure.

Following the initial analyses, normality tests were conducted, leading to the removal of two outliers. This resulted in a normal distribution of the reactivity variables: SBP (*W* = 0.99, *p* = 0.37), DBP (*W* = 0.99, *p* = 0.97), and HR (*W* = 0.98, *p* = 0.13). Subsequently, linear regressions were performed with a two-step approach. In the first step, the variables BMI, gender, and baseline cardiac variables (SBP, DBP, HR) were controlled. Then, in the second step, the psychological variables of optimism, pessimism, negative affect, and positive affect were included. Controlling for the effect of baseline reactivity variables, gender and BMI, a statistically significant effect was found between pessimism and SBP (*β* = −1.07, *t* = −3.258, *p* = 0.002, $\eta_{p}^{2}$ = 0.09) suggesting that higher levels of pessimism decreased SBP cardiac reaction. The complete model explained 17% of the variance of the SBP reactivity, however the other psychological variables did not contribute to the model fit. Regarding DPB reactivity, there is a statistically significant effect of the variable optimism (*β* = 0.504, *t* = 2.303, *p* = 0.0023, $\eta_{p}^{2}$ = 0.08) that increases DBP, and pessimism (*β* = −0.528, *t* = −2.121, *p* = 0.036, $\eta_{p}^{2}$ = 0.06) that reduces it. The model explained 25% of the variance of DBP reactivity scores; the other psychological variables did not present a statistically significant effect. No statistically significant relationships were found between the psychological variables and HR after controlling for the effect of the variables in step 1. The complete results can be found in Table 2.

**Table 2:** Stepwise linear regression models predicting SBP, DBP and HR.

	β	*t*	*p*	$\eta_{p}^{2}$
**Step 1 SBP reactivity**				
Baseline SBP	−0.150	−1.969	0.052	0.00
Sex	−4.483	−2.958	0.004	0.06
BMI	−0.026	−0.168	0.867	0.00
**Step 2**				
Optimism	0.336	1.145	0.255	0.00
Pessimism	−1.074	−3.258	0.002	0.09
Positive affect	−0.135	−1.295	0.199	0.01
Negative affect	0.095	1.142	0.256	0.01
**Step 1 DBP reactivity**				
Baseline DBP	−0.232	−3.581	0.001	0.13
Sex	−1.313	−1.390	0.168	0.01
BMI	0.038	0.328	0.744	0.00
**Step 2**				
Optimism	0.504	2.303	0.023	0.08
Pessimism	−0.528	−2.121	0.036	0.06
Positive affect	−0.022	−0.286	0.775	0.00
Negative affect	−0.009	−0.150	0.881	0.00
**Step 1 HR reactivity**				
Baseline HR	−0.222	−4.571	0.000	0.18
Sex	1.626	1.349	0.181	0.01
BMI	−0.001	−0.005	0.996	0.00
**Step 2**				
Optimism	−0.131	−0.478	0.634	0.00
Pessimism	−0.016	−0.052	0.958	0.00
Positive affect	0.146	1.518	0.132	0.02
Negative affect	−0.033	−0.427	0.671	0.00

## Discussion

The objective of the present study was to examine the relationship between optimism, pessimism, and CVR to stress in young adults, while controlling for variables such as BMI, sex, and positive and negative affect. A statistically significant inverse relationship was found between pessimism and cardiovascular reactivity in both SBP and DBP. Optimism was found to be directly related to DBP. No effect was found on HR in general.

The results suggest a relationship between higher levels of pessimism and reduced CVR to stress in terms of SBP and DBP which aligns with prior research that has found that pessimism could predict diminished CVR (Felt et al., 2020). This dampened cardiac response among individuals with pessimistic tendencies might stem from their inclination to respond less effectively to stressors, often succumbing to challenges or maintaining low expectations of overcoming obstacles (Scheier et al., 1986; Baumgartner et al., 2018; Bajaj et al., 2019). Furthermore, this type of blunted cardiac reaction has been linked to conditions such as depression, the use of psychotropic substances, and overall poorer health (Allen, 2013). Therefore, it appears that pessimism may act as a risk factor for abnormally low or blunted cardiovascular reactions, which could contribute to the heightened incidence of cardiac issues observed in pessimistic individuals (Pänkäläinen et al., 2016; Lee et al., 2019; Whitfield et al., 2020; Krittanawong et al., 2023).

These observations might be because pessimism tends to involve an avoidant or repressive approach to handling stressors, which consequently translates into a defeated attitude when confronted with stressful events (Bajaj et al., 2019). It’s noteworthy that this effect persists even when negative affect is controlled for, possibly indicating that holding negative expectations about the future is detrimental to cardiac health. Alternative explanations for the observed results is that individuals in the sample with pessimistic tendencies might have less motivation to engage fully in the experiment and may not exert maximum effort during the experimental session.

As for optimism, prior research has highlighted its protective role in mitigating the impact of stress on cardiovascular reactivity (Gallagher et al., 2014; Bajaj et al., 2019). However, this study observed an effect of optimism solely on DBP reactivity, and this effect persisted even after accounting for variables such as positive affect, negative affect, BMI, and sex. This suggests that maintaining positive expectations about future events is related to CVR, even when the influence of affect is considered. This implies that the influence of positive expectations on CVR remains intact even when accounting for the effects of positive emotions on health (Chida and Hamer, 2008; Chida and Steptoe, 2008), healthier lifestyles (Steptoe, 2019), physical activity, and sleep. One can hypothesize that this is due to optimism’s tendency to promote approach-oriented problem-solving during times of stress, which in turn might have a protective effect at the psychological level by mobilizing resources for direct coping with stressors (Lee et al., 2019). Optimism prompts individuals to view stressful events as temporary challenges with attainable solutions, resulting in a reduced impact. Therefore, optimism might influence cardiovascular reactivity not solely due to fostering a healthy lifestyle but also through its impact on psychological well-being by fostering a more optimistic outlook on the future. Furthermore, it’s plausible that the subgroup of individuals with high levels of optimism in the sample were more motivated to exert their best effort during the experimental session, thereby contributing to an increase in their cardiovascular reactivity (CVR).

It’s important to emphasized caution when interpreting these results. While our findings are consistent with previous studies that have employed similar procedures, its crucial to acknowledge the existence of studies that have failed to found a relationship between laboratory reactivity measures and those observed in everyday life (Gerin et al., 1998; Baucom et al., 2018; De Calheiros Velozo et al., 2023). This potential disparity could impact the external validity of our current study. Nevertheless, it is worth noting that some investigations have suggested that once the influence of confounding variables in daily life, such as mood, the frequency of adverse events, and stress, are properly controlled for, a relationship with laboratory reactivity measures is found (Kamarck et al., 2003; Schwerdtfeger et al., 2014).

In the context of our present research, the discovery of a positive impact of optimism and a negative impact of pessimism aligns with existing literature (Puig-Perez et al., 2017; Baumgartner et al., 2018; Bajaj et al., 2019). Nonetheless, we advise caution in interpreting the results of our study until further research establishes a robust connection between optimism and reactivity measures obtained in real-world daily scenarios.

In general, positive affect and positive emotions (states of activation and happiness) have been associated with elevated levels of cardiovascular reactivity, while negative affect and states of low activation (such as sadness and anger) have related to diminished reactivity (Pressman et al., 2019). This study’s significance lies in the fact that even after accounting for the influence of emotions, both optimism and pessimism seem to relate to cardiac reactivity. This highlights the significance of positive psychological factors in examining physical and cardiovascular well-being.

The main limitation of this study is the use of psychological tests for measuring optimism and positive affect, which could be influenced by social desirability bias. However, in psychological research, it is common to utilize self-reported tools like psychological tests (Rosenman et al., 2011; Althubaiti, 2016), and the anonymity provided during participation in this study may have helped mitigate the effects of social desirability. Additionally, some variables were not considered in the procedure, such as the potential impact of the circadian cycle on reactivity, unintended stress effects from control variables like recording volume, and the use of the cardboard box to limit foot movement. Additionally, there are other types of stressful stimuli, such as social stimuli, which could potentially yield different results compared to the cognitive task employed in this study.

Another limitation stems from potential sample bias due to the voluntary nature of participation. It’s conceivable that the individuals who chose to participate possessed higher motivation levels, thereby affecting the generalizability of the findings. Furthermore, the results obtained here were derived from a sample of young adults, so they are only applicable to this population. Additionally, the sample size presents a limitation. While the present study involved 107 participants, employing a larger sample could have allowed for the detection of more substantial effects. Nonetheless, due to constraints in time and resources for this research, expanding the sample size was not a viable option. Addressing this limitation, research teams with more extensive resources could replicate the study with a larger participant pool, as outlined in this study’s description.

A significant advantage of the current investigation was the strict control of the sampling sessions, keeping the cardiovascular reactivity measurements as accurate as possible by creating a neutral environment for data collection. In conclusion, this study contributes to the understanding of the role of optimism and pessimism in cardiovascular reactivity to stress and highlights the importance of considering psychological factors in relation to physical health outcomes. Future lines of research could focus on whether optimism has a relationship with other variables that affect cardiovascular reactivity such as personality, negative life events, and other positive emotion variables such as gratitude. Additionally, replicating the analysis with varied forms of stress-inducing stimuli, emphasizing social tasks over cognitive tasks, and involving different age groups would also be important avenues to explore.

## Data availability statement

The raw data supporting the conclusions of this article will be made available by the authors, without undue reservation.

## Ethics statement

The studies involving humans were approved by CEISH Pontificia Universidad Católica del Ecuador (Code: EO-115-2022). The studies were conducted in accordance with the local legislation and institutional requirements. The participants provided their written informed consent to participate in this study.

## Author contributions

CP-G design of the investigation, acquisition and analysis of the data, writing and drafting of the paper. CH-B design of the paper, writing, drafting and revision of the paper. All authors contributed to the article and approved the submitted version.
